# Conceptualization of functional single nucleotide polymorphisms of polycystic ovarian syndrome genes: an in silico approach

**DOI:** 10.1007/s40618-021-01498-4

**Published:** 2021-01-27

**Authors:** B. N. Prabhu, S. H. Kanchamreddy, A. R. Sharma, S. K. Bhat, P. V. Bhat, S. P. Kabekkodu, K. Satyamoorthy, P. S. Rai

**Affiliations:** 1 Department of Biotechnology, Manipal School of Life Sciences, MAHE, Manipal, Karnataka India; 2 Department of Obstetrics and Gynaecology, Dr. T.M.A Pai Hospital, MMMC, MAHE, Manipal, Karnataka India; 3 Department of Cell and Molecular Biology, Manipal School of Life Sciences, MAHE, Manipal, Karnataka India

**Keywords:** Polycystic ovarian syndrome, Single nucleotide polymorphisms, miRNAs, Transcription factors, Enhancers

## Abstract

**Purpose:**

Polycystic ovarian syndrome (PCOS) is a multi-faceted endocrinopathy frequently observed in reproductive-aged females, causing infertility. Cumulative evidence revealed that genetic and epigenetic variations, along with environmental factors, were linked with PCOS. Deciphering the molecular pathways of PCOS is quite complicated due to the availability of limited molecular information. Hence, to explore the influence of genetic variations in PCOS, we mapped the GWAS genes and performed a computational analysis to identify the SNPs and their impact on the coding and non-coding sequences.

**Methods:**

The causative genes of PCOS were searched using the GWAS catalog, and pathway analysis was performed using ClueGO. SNPs were extracted using an Ensembl genome browser, and missense variants were shortlisted. Further, the native and mutant forms of the deleterious SNPs were modeled using I-TASSER, Swiss-PdbViewer, and PyMOL. MirSNP, PolymiRTS, miRNASNP3, and SNP2TFBS, SNPInspector databases were used to find SNPs in the miRNA binding site and transcription factor binding site (TFBS), respectively. EnhancerDB and HaploReg were used to characterize enhancer SNPs. Linkage Disequilibrium (LD) analysis was performed using LDlink.

**Results:**

25 PCOS genes showed interaction with 18 pathways. 7 SNPs were predicted to be deleterious using different pathogenicity predictions. 4 SNPs were found in the miRNA target site, TFBS, and enhancer sites and were in LD with reported PCOS GWAS SNPs.

**Conclusion:**

Computational analysis of SNPs residing in PCOS genes may provide insight into complex molecular interactions among genes involved in PCOS pathophysiology. It may also aid in determining the causal variants and consequently contributing to predicting disease strategies.

**Supplementary Information:**

The online version contains supplementary material available at 10.1007/s40618-021-01498-4.

## Introduction

Polycystic ovarian syndrome (PCOS) is a multifactorial endocrine disorder with uncertain etiologies among reproductive-aged females and is a frequent cause of infertility in women [1]. It is manifested by several endocrine disturbances such as chronic anovulation, hyperandrogenism characterized by frontal alopecia, acne and hirsutism, presence of multiple cysts in ovaries, and metabolic consequences including a high risk of obesity, insulin resistance, type 2 diabetes mellitus (T2DM) and cardiovascular diseases [2, 3] and psychological complications such as increased distress and depression [4]. Although not understood completely, this complex disorder is considered to be caused due to intricate interplay between various factors such as genetic and epigenetic predisposition, ethnicity, environmental influences, and lifestyle [5]. It was also conferred as an evolutionary paradox for impairing fertility in women without diminishing in disease prevalence. Earlier reports on evolutionary dynamics in PCOS encompass only females and not the male's role in the genotype/phenotype distinction. As this disease is known to affect only females, yet males might be the carrier of PCOS linked features such as hyperandrogenism and may contribute to conserving the genetics predisposing to PCOS [6, 7]. Further, these factors can significantly influence the phenotypic complexity of the syndrome.

The pathophysiology of PCOS is relatively challenging due to the involvement of numerous pathways such as insulin signaling pathway, androgen synthesis, altered gonadotropin ratios, glucose, and lipid metabolism [8]. Despite the challenge of the multifaceted nature of PCOS, the heritable factors, including genes and their interaction, gene-environment relation, epigenetic modifications, alteration in proteins, and metabolites, have been reported through different approaches such as genomics, transcriptomics, proteomics, and metabolomics to delineate the molecular pathomechanisms of PCOS [9]. Since the significant information in this complex endocrinopathy is inadequate; there is a prerequisite to integrate the data from Genome-Wide Association Study (GWAS) with in silico analysis.

A gene and its products are controlled by numerous mechanisms that comprise interaction between various genes, pathways, and factors [10]. The most predominant form of genomic variation is Single-nucleotide polymorphisms (SNPs), where two substitute bases exist at a noticeable frequency in humans [11]. Researchers were accustomed to focusing on the SNPs in the coding region of the genome, particularly non-synonymous SNPs (nsSNPs), as they are expected to significantly change the function of encoded proteins [12]. Besides, the unpredicted discovery of the GWAS revealed that > 90% of disease-linked SNPs reside in the non-coding sequence, which is also responsible for contributing to complex diseases [11], and confirms that SNPs can serve as a valuable biomarker to investigate the heritability that influences individuals to specific phenotype including diseases [10]. In the present study, we intended to determine the impact of SNPs in the selected GWAS genes using bioinformatics tools and evaluate their detrimental effects on the structure and function of a protein, miRNA controllers, transcription factor binding elements, and enhancers, which may play a critical role in PCOS susceptibility and assist in delineating the precise pathomechanisms of PCOS.

## Methods

### Identification of genes involved in the pathogenesis of PCOS

A comprehensive literature screening was conducted using the GWAS catalog (https://www.ebi.ac.uk/gwas/). A manual curation procedure was implemented using the search key term "polycystic ovary syndrome" to identify the causative genes at genome-wide significance (P < 5 × 10E^−8^) involved in PCOS pathogenesis.

### Pathway interaction among PCOS genes

The identified PCOS GWAS genes were imported to the Cytoscape tool, and a plug-in named ClueGO v2.5.7 [13] was used for biological and functional interpretation of a large number of genes to constitute the networks. Molecular function, cellular components, biological process, KEGG, and reactome pathways were the different ontologies used in the framework. Kappa statistics were used to connect the terms, and the network was visualized in the circular layout.

### Data retrieval and SNPs characterization

The identified genes and their symbols were subjected to SNP search in the Ensembl genome browser (m.ensembl.org) using the option variant table. The list of SNPs identified was further categorized into 5′-UTR SNPs, synonymous SNPs, intronic SNPs, missense SNPs, 3′-UTR SNPs, splice region SNPs, splice donor SNPs, splice acceptor SNPs, stop retained SNPs, stop-gained SNPs, stop-lost SNPs, and non-coding transcript exon SNPs. Among these SNPs, nonsynonymous SNPs (nsSNPs) were subsequently used for downstream analysis.

### Prediction of nsSNP functional impacts by in silico analysis

The retrieved nsSNP were analyzed using six different tools with mutation score available in the Ensembl genome browser, namely PolyPhen-2 (Polymorphism Phenotyping), SIFT (Sorting Intolerant from Tolerant), CADD (Combined Annotation-Dependent Depletion), Revel (Rare exome variant ensemble learner), MetaLR, and Mutation assessor. Finally, the SNPs categorized as “deleterious” in all 6 tools were selected and analyzed to influence the protein structure and stability.

### Protein modeling and impact of the mutation on protein structure

The native and mutant forms of deleterious SNPs were modeled to predict the mutation’s effect on protein structure and function. We tabulated the hydropathy index proposed by Jack Kyte and Russell F Doolittle [14], which revealed the modification in hydrophilicity or hydrophobicity due to amino acid change in the protein. The proteins structures were computed using Iterative Threading ASSEmbly Refinement (I-TASSER) [15] using an amino acid template from the Uniprot database. Further mutation analysis and energy calculations were performed on the Swiss-Pdb viewer. PyMOL software’s align function was used to calculate the root-mean-square deviation (RMSD) value of mutant type from native protein.

### Functional microRNA target SNPs prediction

The identified genes involved in PCOS pathogenesis were subjected to functional microRNA binding SNP prediction using the miRNA-related SNPs (MirSNP) database [16], the PolymiRTS database [17], and the microRNA related Single Nucleotide Polymorphisms v3 (miRNASNP3) database [18]. The gene symbols of the shortlisted genes were used in the MirSNP database to search the miRNA binding SNP sites and their effects on the target site. In the PolymiRTS database, the search options containing gene symbol was used to retrieve the SNPs and their associated miRNAs at ancestral and mutant allele. The miRNASNP3 database was used to retrieve microRNA related SNPs with their impact on the target gain/loss in the 3′-UTR region.

### SNPs at transcription factor binding site

The identified PCOS genes were utilized to find the SNPs in transcription factor binding sites using SNP2TFBS [19]. The annotated variant option was used to retrieve the SNPs present in the 5′-UTR and upstream regions. The SNPInspector (trail access version) in Genomatix Software Suite (https://www.genomatix.de/) was used to predict whether SNPs in TFBS create or disrupt the transcription factor binding sites.

### SNPs in enhancers

The identified GWAS genes at genome-wide significance in PCOS were used to examine the impact of SNPs in enhancers using EnhancerDB [20] and HaploReg v4.1, which is developed by ENCODE laboratories [21]. The search option containing gene was used in the EnhancerDB database to search the SNPs located in the enhancers of the respective genes, and the regulatory motifs that were altered of those SNPs were reported using HaploReg.

### Linkage disequilibrium analysis of functional SNPs

The identified SNPs that may be functional, obtained by analysing SNPs in coding region, 3′-UTR, 5′-UTR, upstream region and introns of selected GWAS genes in PCOS were further evaluated by performing Linkage disequilibrium (LD) analysis. These SNPs were further correlated with reported PCOS GWAS SNPs using LDlink [22] to examine their impact on disease progression.

## Results

### Identification of genes associated with the pathogenesis of PCOS

**Fig. 1:**
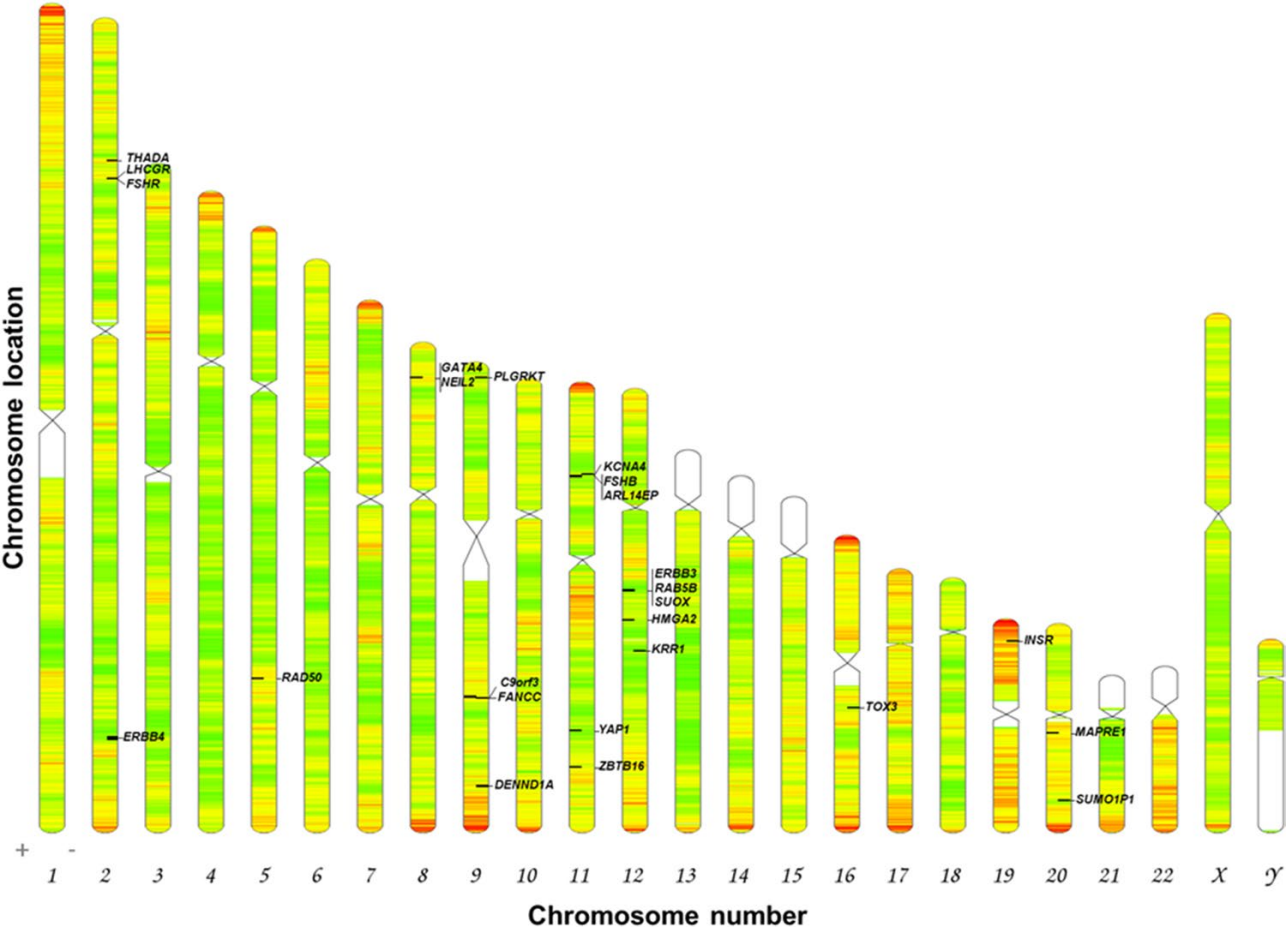
Chromosome-wide distribution of PCOS GWAS genes

**Fig. 2:**
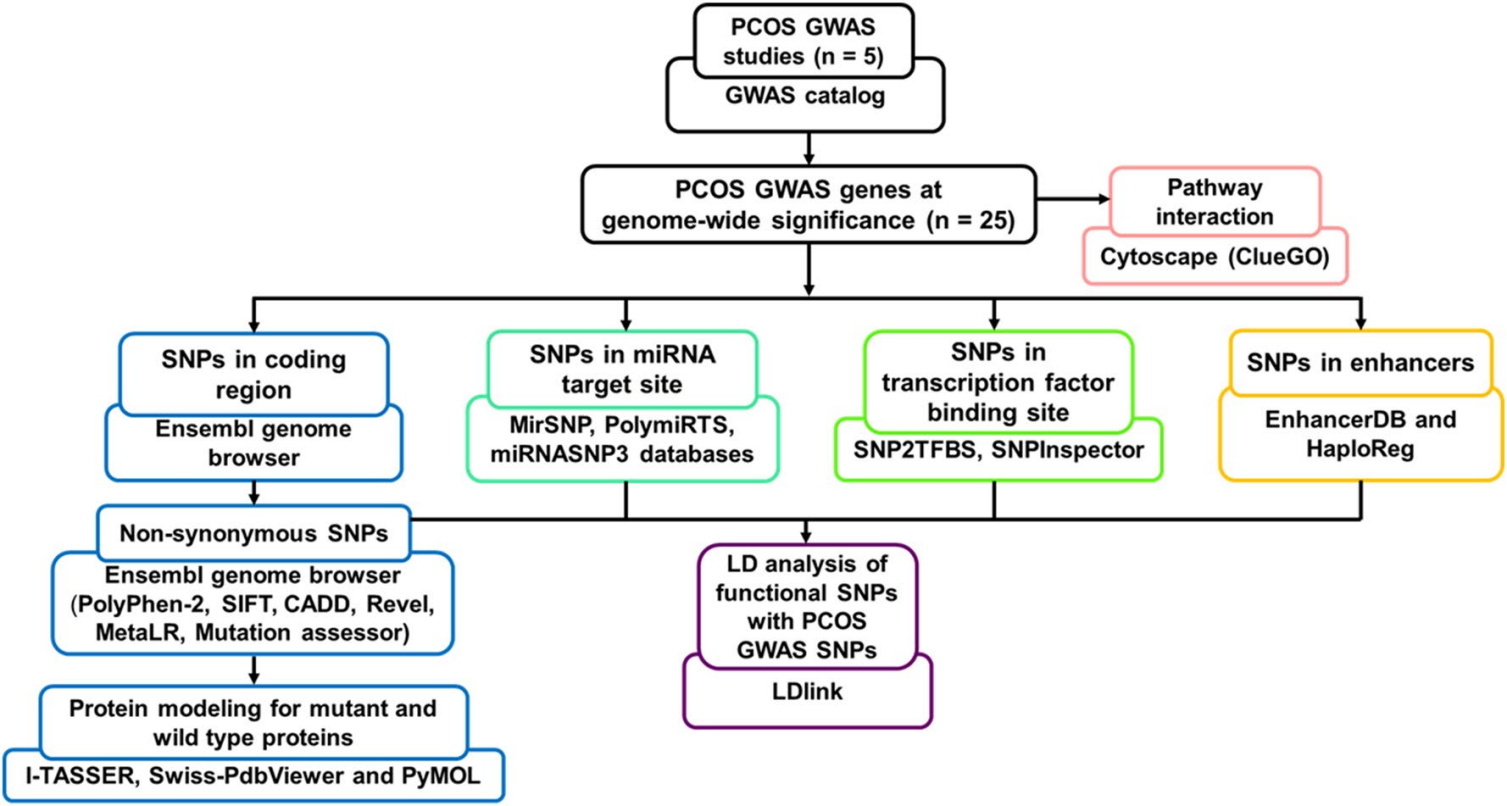
Schematic representation of in silico workflow

We shortlisted 25 GWAS genes linked with PCOS pathogenesis. The details of the genome-wide significant SNPs used to identify the in/nearest genes associated with PCOS were tabulated from the reported studies (Online Resource 1, 2). The shortlisted genes were mapped them using Idiographica. The representation showed the distribution of genes across 9 autosomes including chromosome 2, 5, 8, 9, 11, 12, 16, 19, 20 all over the genome (Fig. 1). The schematic representation of in silico workflow is depicted in the Fig. 2 (Fig. 2).

### Pathway interaction among PCOS genes

**Fig. 3:**
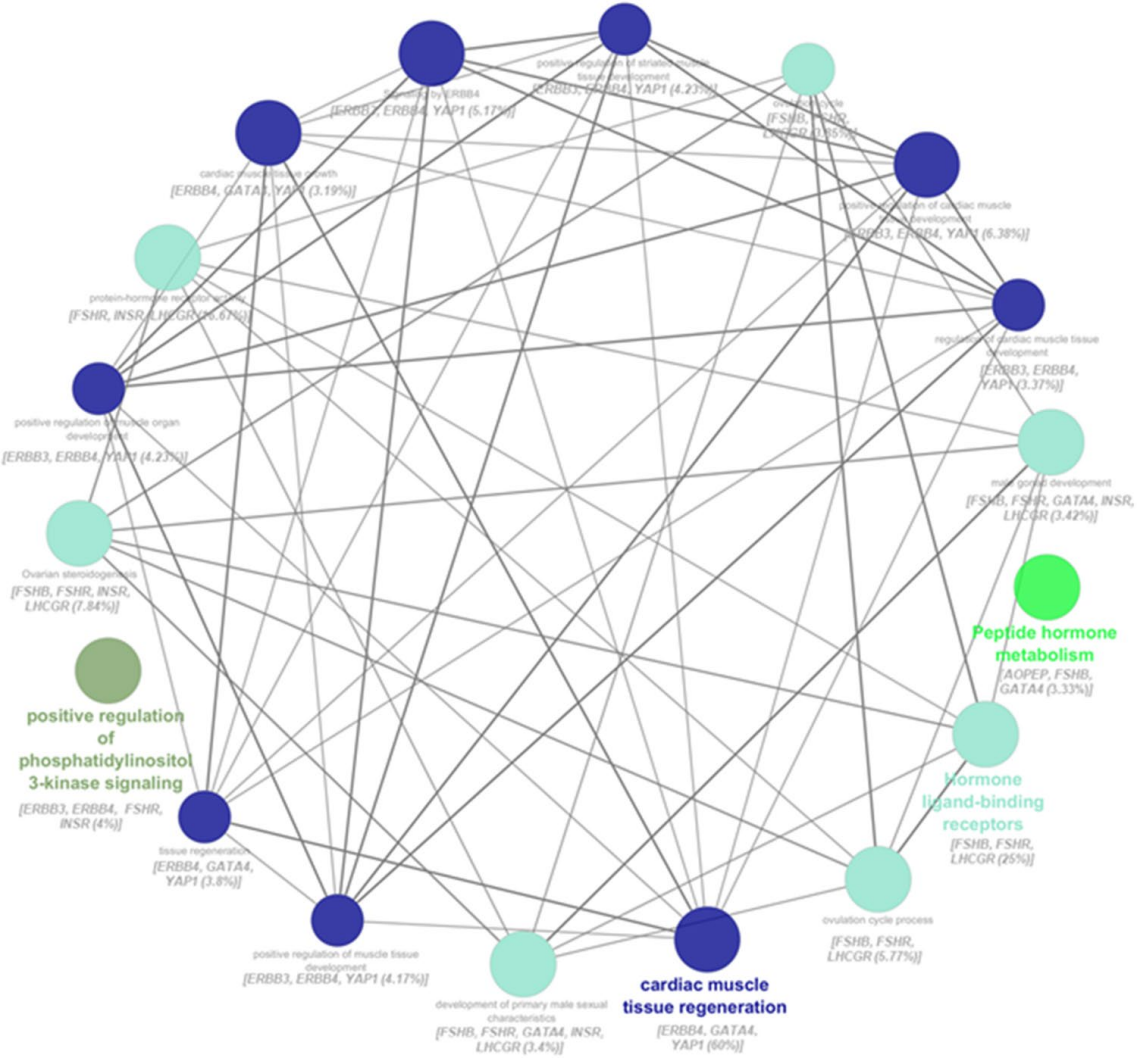
Pathway interaction of PCOS genes

The association between PCOS genes using the molecular function, cellular components, biological process, KEGG, and reactome pathways displayed a network showing the interaction of 9 out of 25 shortlisted genes and their pathways after performing enrichment/depletion (Two-sided hypergeometric test) (Fig. 3). The framework also showed 4 Kappa score groups such as hormone ligand-binding receptors, peptide hormone metabolism, cardiac muscle tissue regeneration, and positive regulation of phosphatidylinositol 3-kinase signaling (Fig. 3). It was found that *ERBB4, GATA4* and*, YAP1* genes contributed 60 percent in cardiac muscle tissue regeneration (Fig. 3).

### Characterization of SNPs

**Fig. 4:**
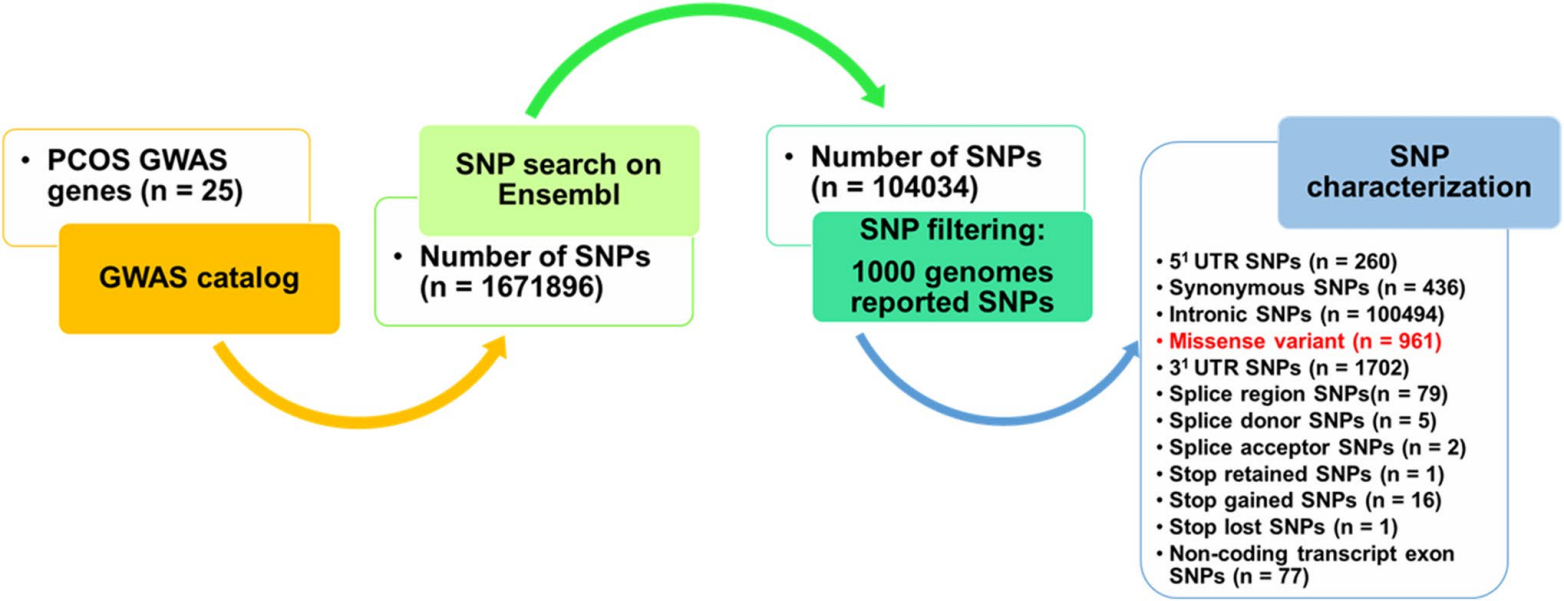
Schematic representation of in silico SNP search and characterization

**Fig. 5:**
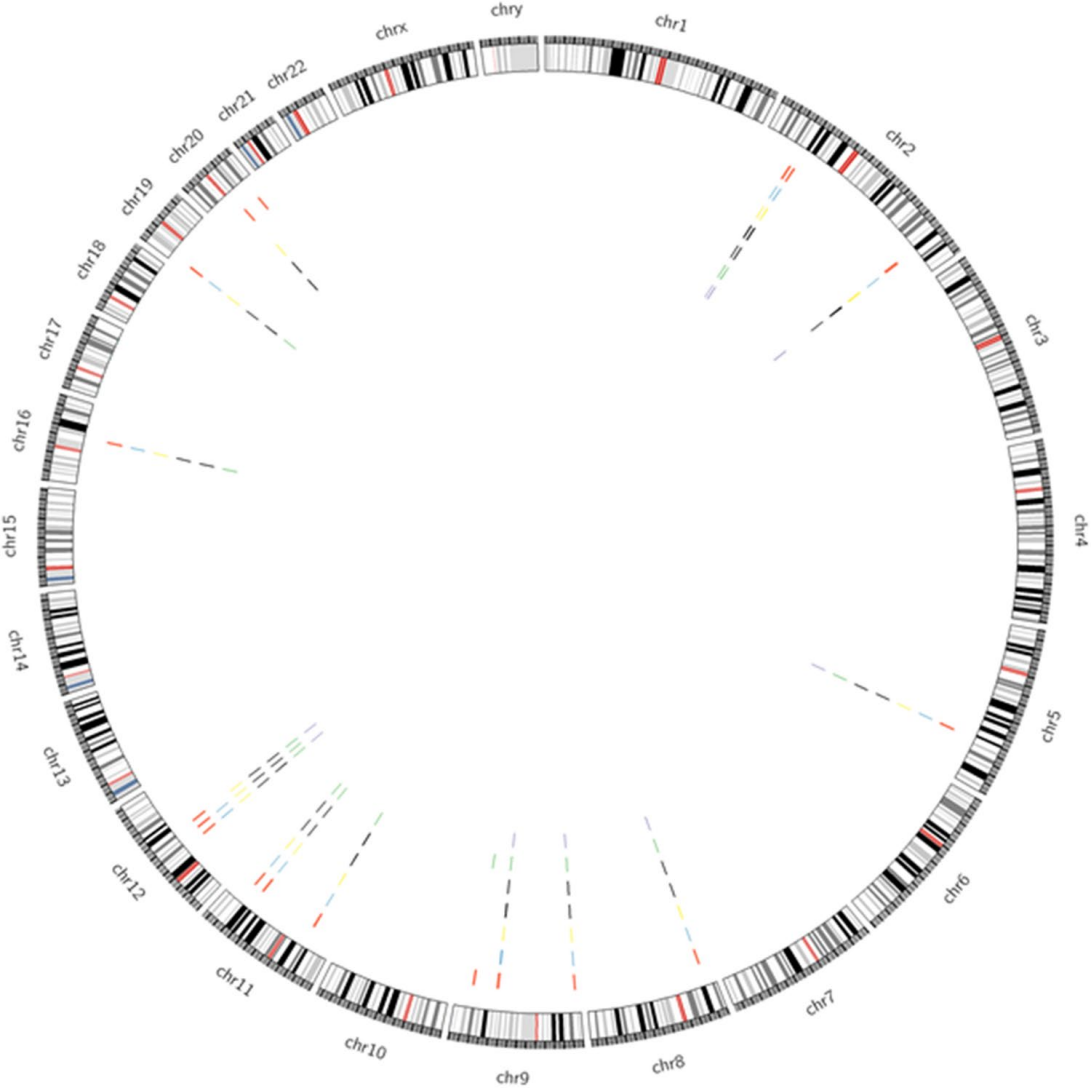
Circos plot representing SNP distribution across 25 genes involved in PCOS pathogenesis showing (outer ring) all the chromosomes, 25 genes (from outer ring inwards), 5′-UTR SNPs, synonymous SNPs, missense variants, 3′-UTR SNPs, splice variants (splice region, splice donor, splice acceptor), inner most ring constitutes stop retained, stop-gained and stop-lost SNPs

A total of 16,71,896 SNPs were retrieved by a search using the Ensembl genome browser (GRCh38.p13). As 1000 Genomes Project was recognized with ample account of genetic variations in humans, these SNPs were filtered for the 1000 Genomes Project lead to the identification of 1,04,034 SNPs. Further, these SNPs were categorized based on their function. 260 SNPs were present in the 5′-UTR region, 436 were synonymous SNPs, 1,00,494 were intronic SNPs, 1702 were 3′-UTR SNPs, 86 were splice variants (splice region, splice donor, splice acceptor), 1 stop retained SNP, 16 stop-gained SNPs, 1 stop-lost SNP, 77 were non-coding transcript exon SNPs, and 961 were missense variants of the genes involved in the PCOS (Figs. 4, 5).

### Selection of deleterious nsSNPs

**Fig. 6:**
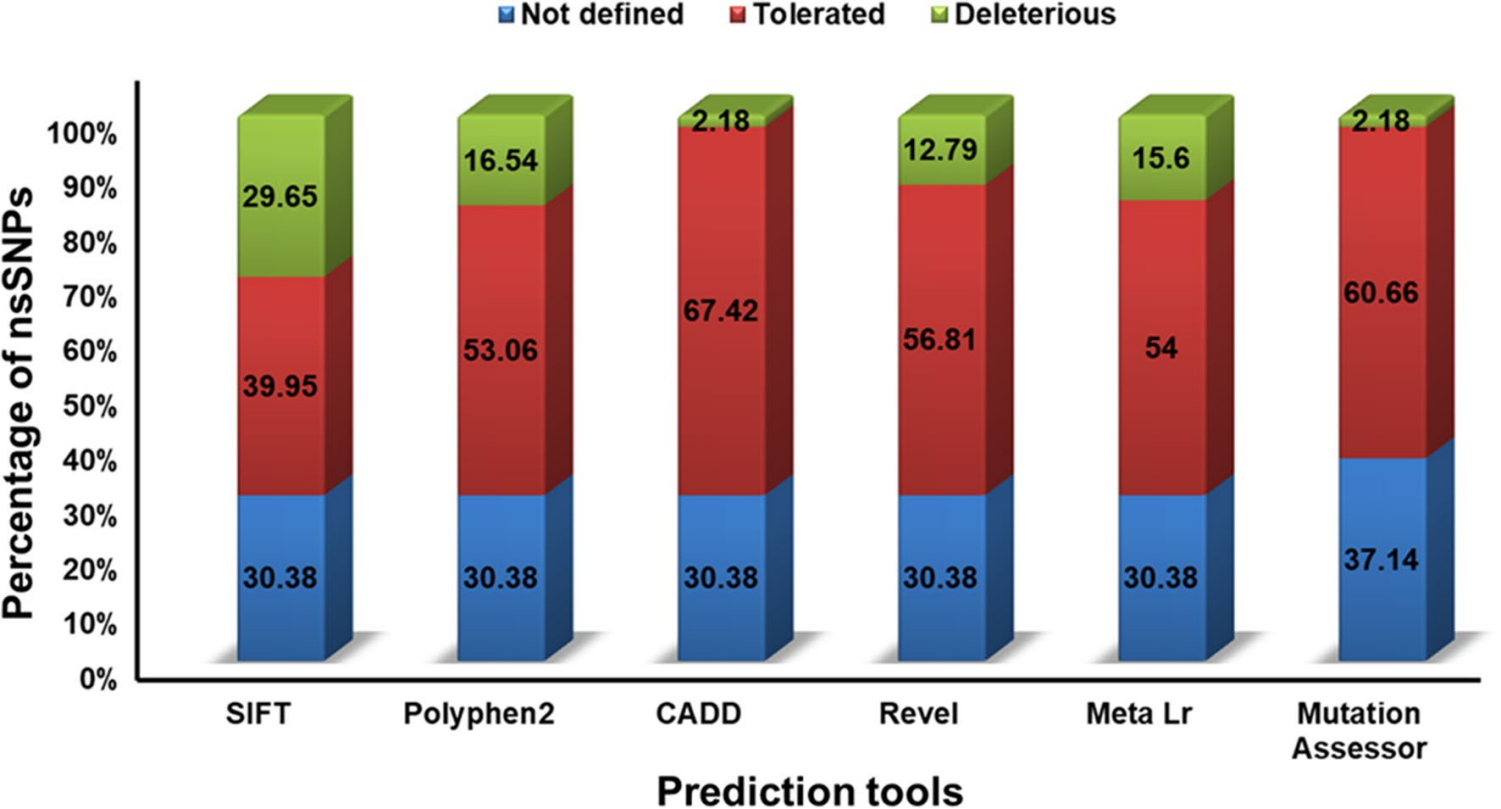
Functional characterization of SNPs in PCOS genes

**Fig. 7:**
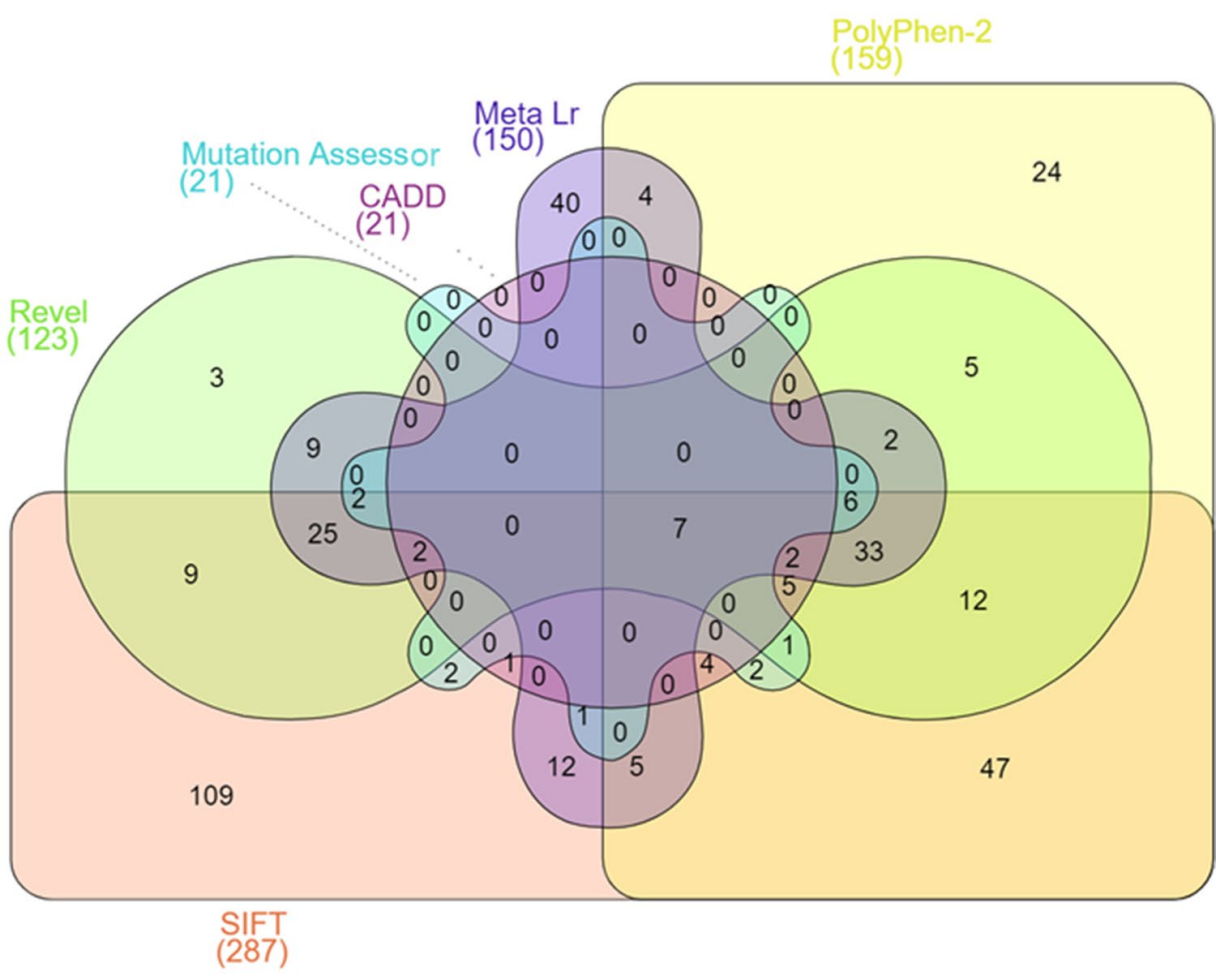
Functional prediction of common non-synonymous SNPs by six pathogenicity predictions

Among 961 missense variants, 285 (29.65%) were reported as “deleterious” by SIFT, while the frequency of mutation was reduced to 159 (16.54%) as “probably damaging” by PolyPhen-2, 21 (2.18%) as “likely deleterious” were analysed by CADD, and 123 (12.79%) as “likely disease-causing” by Revel, 150 (15.60%) as “damaging” by Meta LR and 21 (2.18%) as “high” by Mutation Assessor (Fig. 6). Six different bioinformatic tools (SIFT, PolyPhen-2, CADD, Revel, Meta LR, Mutation Assessor) collectively highlighted 7 deleterious nsSNPs (Fig. 7) which included *ERBB4* rs192066345 and rs528780505, *GATA4* rs180765750, *INSR* rs79312957, *LHCGR* rs121912525, *SUOX* rs575660698, and *YAP1* rs199505545 (Online Resource 3).

### Protein modeling and impact of the mutation on protein structure

**Fig. 8:**
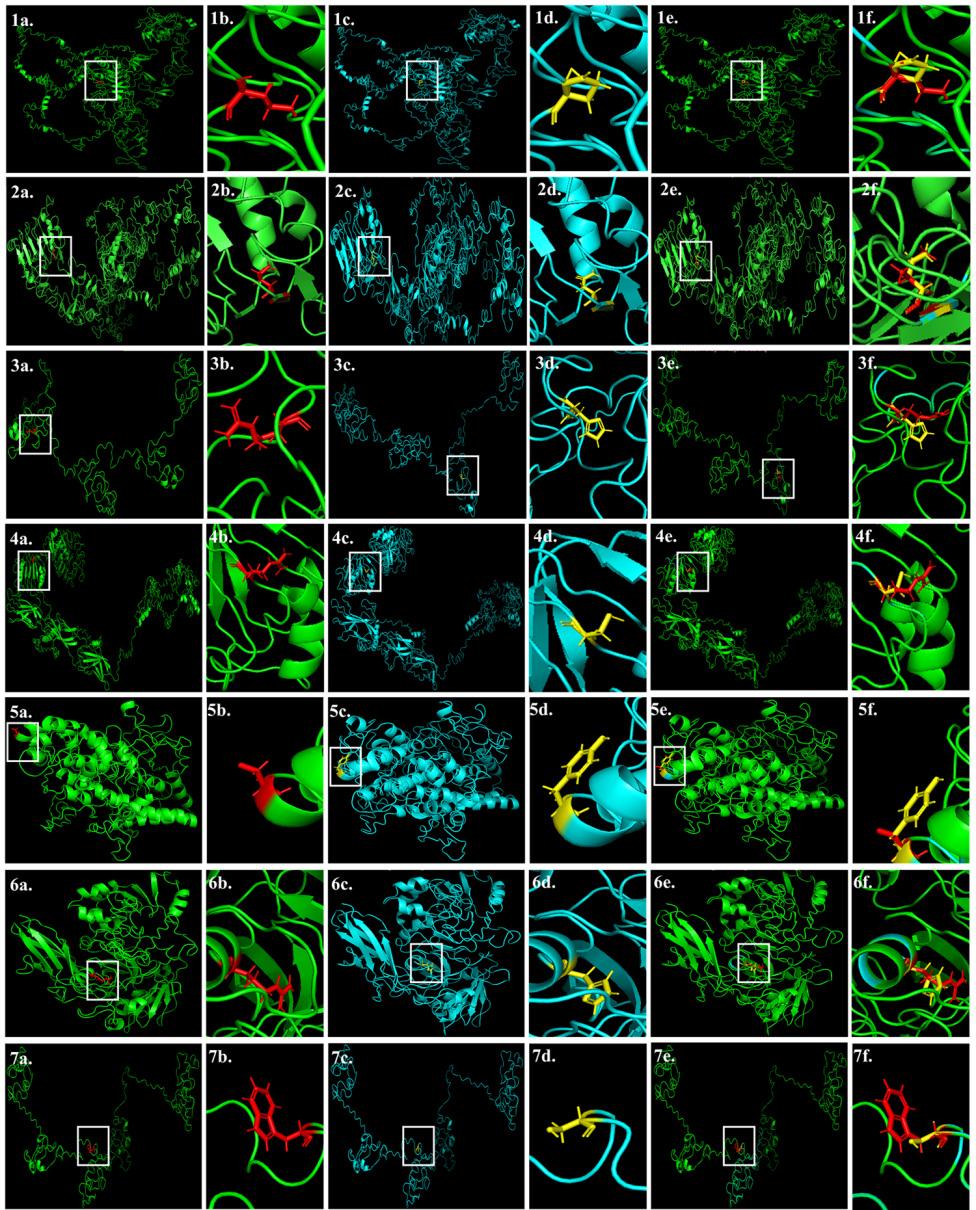
Native, mutant, and superimposition of native and mutant modeled structures of the *ERBB4* (1) rs192066345 and (2) rs528780505, (3) *GATA4* rs180765750, (4) *INSR* rs79312957, (5) *LHCGR* rs121912525, (6) *SUOX* rs575660698, and (7) *YAP1* rs199505545. **a** Structure of native protein. **b** Enlarged structure of native protein (**c**) Structure of mutant protein. **d** Enlarged structure of mutant protein (**e**) Superimposed model of native and mutant protein structures. **f** Enlarged superimposed model of native and mutant protein structures

The structures of the proteins were modelled using I-TASSER (Fig. 8). Out of 7 nsSNPs identified, change in amino acid in ERBB4 (rs528780505) suggested a change in polarity and hydrophobicity/hydrophilicity (Online Resource 4). The polarity and hydropathy index for all the polymorphisms are listed in Online Resource 4. The rs528780505 showed altered amino acid from isoleucine to asparagine at 362nd position, which resulted in a change in polarity from non-polar to polar and the hydropathy index from 4.5 to  − 3.5. There was an observed difference in the total free energy of the wild type (− 33,905.453 kJ/mol) and mutant type (− 34,064 kJ/mol) protein (Online Resource 5). The root-mean-square deviation calculated between the wild types and mutants was 0.001 Ǻ for ERBB4 rs528780505. The RMSD value of all the proteins are tabulated (Online Resource 5).

### Prediction of functional microRNA target SNPs

In the study, we used 3 different tools (MirSNP, PolymiRTS, miRNASNP3) which concordantly showed 3 SNPs (Online Resource 6) in the microRNA target binding sites, namely, rs1042725, rs7312910 in the *HMGA2* gene, and rs242538 in the *MAPRE1* gene with the minor allele frequency (MAF) > 0.1. The table also showed whether miRNAs associated with SNPs within the target site would create or break or decrease or enhance a miRNA-mRNA binding site (Online Resource 6).

### SNPs at transcription factor binding siteSsec2

Using SNP2TFBS, a total of 10 SNPs with MAF > 0.1 were identified in TFBS, out of which 9 SNPs are present in the upstream and 1 SNP in the 5′-UTR region. Among these, SNPInspector predicted that rs8191514 in the *NEIL2* generated a binding site for twenty transcription factors, and rs62579216 in the *DENND1A* gene deleted the binding site for nine transcription factors. The impact of 10 SNPs at TFBS reported whether SNPs would generate or delete the sites for the binding of transcription factors (Online Resource 7).

### SNPs in enhancers

In the present study, we used 2 databases (EnhancerDB and HaploReg), which collectively reported 8 intronic SNPs in the enhancers with MAF > 0.1. Among these, rs11670022 in the *INSR* gene showed 5 altered regulatory motifs which included E2A, HEN1, Lmo2, Myf, ZEB1 followed by rs73488786 in the *INSR* gene had shown 4 altered regulatory motifs namely, AP-1, BDP1, CTCF, SMC3 and rs56394135 in the *RAD50* gene showing 4 altered regulatory motifs namely, Dbx2, Maf, Pou2f2, THAP1. The details of enhancer SNPs and their altered regulatory motifs are tabulated (Online Resource 8).

### Linkage disequilibrium analysis of functional SNPs

Using LDlink, a total of 28 SNPs that may be functional were further examined to correlate with reported PCOS GWAS SNPs. Out of which 4 SNPs were in LD, namely, rs8191514 in the *NEIL2* gene is correlated with rs804279. rs242538 in the *MAPRE1* gene is correlated with rs853854. rs12237685 in the *DENND1A* gene is correlated with rs9696009 and rs2479106. rs3846732 in the *RAD50* gene is correlated with rs13164856. *R*^2^, D′, and *p* value of the selected SNPs with reported PCOS GWAS SNPs were calculated and cataloged (Table 1).

**Table 1:** Linkage disequilibrium analysis showing correlation of functional SNPs with reported PCOS GWAS SNPs

Sl no	Gene	rsID	MAF	Correlation with reported PCOS GWAS SNPs	*R*^2^	D′	*p* value
1	*NEIL2*	rs8191514	0.13	rs804279	0.4	0.97	< 0.0001
2	*MAPRE1*	rs242538	0.19	rs853854	0.69	1	< 0.0001
3	*DENND1A*	rs12237685	0.36	rs9696009	0.18	0.68	< 0.0001
				rs2479106	0.61	0.87	< 0.0001
4	*RAD50*	rs3846732	0.4	rs13164856	0.79	0.97	< 0.0001

*NEIL2* nei like dna glycosylase 2*, MAPRE1* microtubule associated protein rp/eb family member 1, *DENND1A* DENN domain containing 1A, *RAD50* RAD50 double strand break repair protein

## Discussion

Exertions intended to interpret the molecular mechanisms of multifaceted diseases like PCOS are supported by high-throughput approaches to identify genetic variations resulting in the generation of large amounts of data [10]. To manage these vast amounts of data and to provide insight into PCOS development, researchers have used a variety of in silico prediction tools [23]. In the present study, after reviewing publications from the GWAS catalog, the potential causal genes at genome-wide significance were shortlisted and subsequently examined to identify and predict the deleterious SNPs and their impact on disease progression. Prediction of SNPs was made using six different tools, namely, SIFT, PolyPhen-2, CADD, Revel, Meta Lr, and Mutation assessor. The interpretation of these data should be evaluated accurately to address the significance of gene and should be verified whether the genetic variants are deleterious and impact protein structure or not [24]. Hence evaluation of these genetic variations is carefully performed with the use of different SNP prediction tools by selecting the overlapping predictions to mitigate the false-positive interpretation [10].

Our computational approach has identified 7 deleterious nsSNPs from 6 SNP prediction tools. These genetic variations reside in different genes such as *ERBB4, GATA4, INSR, LHCGR, SUOX*, and *YAP1*. So far, minimal investigations have been carried out to predict the effect of nsSNPs. Despite, few studies have been reported the role of *INSR* rs79312957, *LHCGR* rs121912525, in complex traits. An in silico study conducted by Mahmud et al. 2016 identified that mutation in *INSR* (rs79312957) caused type A insulin resistance, which is a prominent feature observed in PCOS females [25]. During adolescence, the type A insulin resistance in PCOS females shows higher insulin levels in the bloodstream which interacts with the different hormones and induce aberrations in menstruation, presence of multiple cysts in the ovaries, and other related features of the syndrome [26]. Interestingly, the mutation in LH receptor (rs121912525) has a higher chance of causing partial ovarian failure manifested by defects in ovarian folliculogenesis, anovular menstruation, luteal phase defects, imperfect feminization at adolescence, amenorrhoea and, infertility in females [27], which are again the characterized features of PCOS.

The effect of nsSNP, rs79312957 in *INSR*, can cause numerous insulin-resistant diseases. An earlier computational study by Mahmud et al*.,* 2016 showed the structural modification between the native and mutant forms of protein INSR rs79312957, based on the value of Gibbs free energy [25]. The variation in free energy, when it deviates from native to mutant type, the variation in free energy indicates protein stability [10]. The authors also provided computational evidence for the destabilizing effect of nsSNP rs79312957 on the insulin receptor which is considered to impact protein structure and function [25]. Hence, we used a structural-based method to determine the influence of 7 deleterious nsSNPs on its protein structure. We have assessed changes in polarity, hydrophobicity/hydrophilicity, and hydropathy index in the present study. Besides, we have also calculated change in energy from native to mutant protein type and RMSD value for all the 7 nsSNPs, which might contribute strength to assess the protein function. Our study also confirms the expected effects of INSR rs79312957 by depicting the deviation of RMSD value from native to the mutant form of protein.

Research on miRNAs has shown that miRNAs binding at the 3′-UTR region silences the genes and is involved in gene regulation at a posttranscriptional level. Also, alterations in the miRNA binding sites can induce impaired binding of the miRNAs affecting its function [10]. The outcome of the GWAS has resulted in the discovery of a massive number of SNPs. Although the impact of SNPs in the noncoding site of the gene is scant, we focussed on 3′-UTR SNPs in the present study. Thus, we retrieved the SNP data of the genes responsible for PCOS pathology to decipher the miRNA sites using MirSNP, PolymiRTS, miRNASNP3 databases and further investigated whether miRNAs associated with SNPs within the target site would create or break or decrease a miRNA-mRNA binding site. In the current approach, LD analysis was performed between selected SNPs that may be functional and PCOS GWAS SNPs to examine their impact on PCOS pathogenesis. LD analysis revealed that *MAPRE1* rs242538 was correlated with the reported GWAS SNP rs853854 (*MAPRE1*) in PCOS (*R*^2^: 0.6, D′: 1, *p* value < 0.0001).

Similarly, the effect of SNPs in TFBS and enhancers were also taken into consideration. SNPs at TFBS possibly affect gene regulation by changing the binding ability of the corresponding TF created by SNP alleles [28]. Our study collectively showed 10 SNPs in the 5′-UTR and upstream region, which controls the expression of genes involved in PCOS. Out of 10 SNPs, rs8191514 in the *NEIL2* gene generated a binding site for twenty transcription factors and was found to be in LD with the reported GWAS SNP rs804279 (*NEIL2*) in PCOS (*R*^2^: 0.4, D′: 0.97, *p* value < 0.0001). Studies have revealed that disease or trait linked non-coding SNPs modify the functions of regulatory motifs, such as enhancers that classically control gene expression [29]. A sum of 8 SNPs in the enhancers with their altered regulatory motifs were identified. Out of which, 2 SNPs were found to be LD with the reported GWAS SNPs in PCOS namely, *DENND1A* rs12237685, *RAD50* rs3846732. Henceforth in the current study, a total of 4 SNPs that were correlated with PCOS GWAS SNPs which implies these linked SNPs would be more likely pathogenic in PCOS than functional SNPs not so linked, thus that are discussed above should be crucially taken into account for delineating the precise pathomechanisms of PCOS.

## Conclusion

In the present in silico analysis, efforts were taken to unveil the remarkable findings to report the genetic markers that regulate the expression of genes to portray the pathomechanisms of PCOS. The use of computational gene mining tactics assists primarily in identifying the causal genes and their interaction in PCOS pathway and aid in evaluating the impact of SNPs in different regions of the gene. The data constitutes a structural foundation to figure out complex molecular connections among genes involved in PCOS pathophysiology and consequently contributes to predicting disease strategies. However, when an SNP is likely linked with a trait or disease, it is commonly assumed that the SNP functions through nearby genes. Hence, it is evident that the current approach may miss some relevant genes. In addition, as we focused on genes, this study will not have identified intronic or intergenic SNPs that contribute to the pathophysiology of PCOS.

## Supplementary Information

Below is the link to the electronic supplementary material.

Supplementary file1 Online Resource 1. Details of shortlisted genes based on genome wide significant SNPs and their chromosome, position, allele frequency, distance between the SNP and the gene, odds ratio, and p-value from the reported PCOS GWAS studies (DOCX 54 KB)

Supplementary file2 Online Resource 2. Details of selected genome wide significant genes for downstream analysis (DOCX 51 KB)

Supplementary file3 Online Resource 3. Deleterious nsSNPs and associated amino acid change (DOCX 17 KB)

Supplementary file4 Online Resource 4. Polarity and hydrophobicity/hydrophilicity of the reported deleterious nsSNPs (DOCX 15 KB)

Supplementary file5 Online Resource 5. Total energy (wild and mutant type), change in energy and RMSD value of the reported deleterious nsSNPs (DOCX 15 KB)

Supplementary file6 Online Resource 6. miRNA target site SNPs with MAF>0.1 (DOCX 16 KB)

Supplementary file7 Online Resource 7. Impact of SNPs in the transcription factor binding site with MAF>0.1 (DOCX 17 KB)

Supplementary file8 Online Resource 8. SNPs in enhancers and their altered regulatory motifs with MAF>0.1 (DOCX 16 KB)

## Data Availability

The datasets generated during and/or analysed during the current study are available from the corresponding author on reasonable request.
