# Supplementary material for: Conceptualization of functional single nucleotide polymorphisms of polycystic ovarian syndrome genes: an in silico approach
Source: J Endocrinol Invest. 2021 Jan 27;44(8):1783–93. doi: 10.1007/s40618-021-01498-4 (PMC8285346; doi:10.1007/s40618-021-01498-4)
Supplement: Supplementary file 1 — Supplementary file1 Online Resource 1. Details of shortlisted genes based on genome wide significant SNPs and their chromosome, position, allele frequency, distance between the SNP and the gene, odds ratio, and p-value from the reported PCOS GWAS studies (DOCX 54 KB) [file 40618_2021_1498_MOESM1_ESM.docx]

**Online Resource 1.** Details of shortlisted genes based on genome wide significant SNPs and their chromosome position, allele frequency, odds ratio, and p value from the reported PCOS GWAS studies

| **Sl no.** | **Gene** | **SNPs** | **Chromosome position** | **MAF** | **Distance between the SNP**  **and the gene** | **Odds ratio** | ***p* value** | **References** |
| --- | --- | --- | --- | --- | --- | --- | --- | --- |
| 1 | *HMGA2* | rs2272046 | 12:65830681 | 0.02 | Intragenic | 0.7 | 1.95x10^-21^ | [31] |
| 2 | *DENND1A* | rs10818854 | 9:123684499 | 0.06 | Intragenic | 1.51 | 9.40x10^-18^ | [30] |
| 3 | *FSHB* | rs11031006 | 11:30204981 | 0.07 | 26033 bp downstream |  | 1.9x10^-8^ | [32] |
| 4 | *DENND1A* | rs10986105 | 9:123787676 | 0.07 | Intragenic | 1.47 | 6.90x10^-15^ | [30] |
| 5 | *KCNA4 / FSHB* | rs11031006 | 11:30204981 | 0.07 | 187951 bp upstream / 26033 bp downstream |  | 1.9x10^-8^ | [32] |
| 6 | *ARL14EP / FSHB* | rs11031005 | 11:30204809 | 0.07 | 30452 bp downstream / 118295 bp downstream | 0.85 | 8.66x10^-13^ | [34] |
| 7 | *YAP1* | rs11225154 | 11:102172509 | 0.1 | Intragenic | 1.22 | 7.6x10^-11^ | [33] |
| 8 | *THADA* | rs13429458 | 2:43411699 | 0.1 | Intragenic | 0.67 | 1.73x10^-23^ | [30] |
| 9 | *C9orf3* | rs3802457 | 9:94979054 | 0.1 | Intragenic | 0.77 | 5.28x10^-14^ | [31] |
| 10 | *YAP1* | rs1894116 | 11:102199908 | 0.1 | Intragenic | 1.27 | 1.08x10^-22^ | [31] |
| 11 | *RAB5B, SUOX* | rs705702 | 12:55996852 | 0.1 | 169 bp upstream / 4305 bp upstream | 1.27 | 8.64x10^-26^ | [31] |
| 12 | *ERBB4* | rs2178575 | 2:212527042 | 0.1 | Intragenic | 1.18 | 3.34x10^-14^ | [34] |
| 13 | *DENND1A* | rs9696009 | 9:123856954 | 0.1 | Intragenic | 1.22 | 7.96x10^-11^ | [34] |
| 14 | *ZBTB16* | rs1784692 | 11:114078510 | 0.1 | Intragenic | 1.15 | 1.88x10^-10^ | [34] |
| 15 | *LHCGR* | rs13405728 | 2:48751020 | 0.2 | Intragenic | 0.71 | 7.55x10^-21^ | [30] |
| 16 | *C9orf3* | rs4385527 | 9:94886305 | 0.2 | Intragenic | 0.84 | 5.87x10^-9^ | [31] |
| 17 | *GATA4 / NEIL2* | rs804279 | 8:11766380 | 0.2 | 6378 bp upstream / 3259 bp downstream |  | 8.0x10^-10^ | [32] |
| 18 | *C9orf3* | rs10993397 | 9:94917489 | 0.2 | Intragenic |  | 4.6x10^-13^ | [32] |
| 19 | *TOX3* | rs8043701 | 16:52341865 | 0.2 | Intragenic | 0.88 | 9.61x10^-10^ | [34] |
| 20 | *MAPRE1* | rs853854 | 20:32832951 | 0.2 | Intragenic | 0.91 | 2.36x10^-9^ | [34] |
| 21 | *ERBB4* | rs1351592 | 2:212529988 | 0.3 | Intragenic | 1.18 | 1.2x10^-12^ | [33] |
| 22 | *RAD50* | rs13164856 | 5:132477512 | 0.3 | Intragenic | 1.13 | 3.5x10^-9^ | [33] |
| 23 | *KRR1* | rs1275468 | 12:75541377 | 0.3 | Intragenic | 1.13 | 1.9x10^-8^ | [33] |
| 24 | *FSHR* | rs2349415 | 2:49020693 | 0.3 | Intragenic | 1.19 | 2.35x10^-12^ | [31] |
| 25 | *TOX3* | rs4784165 | 16:52313907 | 0.3 | Intragenic | 1.15 | 3.64x10^-11^ | [31] |
| 26 | *INSR* | rs2059807 | 19:7166098 | 0.3 | Intragenic | 1.14 | 1.09x10^-8^ | [31] |
| 27 | *PLGRKT* | rs10739076 | 9:5440589 | 0.3 | Intragenic | 1.12 | 2.51x10^-8^ | [34] |
| 28 | *FANCC* | rs7864171 | 9:94960984 | 0.3 | Intragenic | 0.91 | 2.95x10^-8^ | [34] |
| 29 | *KRR1* | rs1795379 | 12:75547262 | 0.3 | Intragenic | 0.89 | 1.81x10^-9^ | [34] |
| 30 | *THADA* | rs7563201 | 2:43334641 | 0.4 | Intragenic | 1.13 | 3.3x10^-10^ | [33] |
| 31 | *THADA* | rs12468394 | 2:43334022 | 0.4 | Intragenic | 0.72 | 1.59x10^-20^ | [30] |
| 32 | *THADA* | rs12478601 | 2:43494369 | 0.4 | Intragenic | 0.72 | 3.48x10^-23^ | [30] |
| 33 | *DENND1A* | rs2479106 | 9:123762933 | 0.4 | Intragenic | 1.34 | 8.12x10^-19^ | [30] |
| 34 | *FSHR* | rs2268361 | 2:48974473 | 0.4 | Intragenic | 0.87 | 9.89x10^-13^ | [31] |
| 35 | *SUMO1P1* | rs6022786 | 20:53830764 | 0.4 | Intragenic | 1.13 | 1.83x10^-9^ | [31] |
| 36 | *ERBB3 / RAB5B* | rs2271194 | 12:56083910 | 0.4 | 7111 bp upstream / 87227 bp upstream | 1.1 | 4.57x10^-9^ | [34] |

*^HMGA2 high-mobility group AT-hook 2, DENND1A^* ^DENN domain containing 1A, FSHB follicle stimulating hormone subunit beta, KCNA4 potassium voltage-gated channel subfamily A member 4,^ *^ARL14EP^* ^ADP ribosylation factor like GTPase 14 effector protein,^ *^YAP1^* ^yes^ ^associated protein 1, THADA thyroid adenoma-associated protein,^ *^C9orf3^* ^chromosome 9 open reading frame 3, RAB5B ras-related protein rab-5b,^ *^SUOX^* ^sulfite oxidase,^ *^ERBB4^* ^erb-b2 receptor tyrosine kinase 4,^ *^ZBTB16^* ^zinc finger and BTB domain containing 16,^ *^LHCGR^* ^luteinizing hormone/choriogonadotropin receptor,^ *^GATA4^* ^GATA binding protein 4,^ *^NEIL2^* ^nei like dna glycosylase 2, TOX3 TOX high mobility group box family member 3,^ *^MAPRE1^* ^microtubule associated protein rp/eb family member 1,^ *^RAD50^* ^RAD50 double strand break repair protein,^ *^KRR1^* ^KRR1 small subunit processome component homolog, FSHR follicle stimulating hormone receptor,^ *^INSR^* ^insulin receptor,^ *^PLGRKT^* ^plasminogen receptor with A c-terminal lysine^*^, FANCC^* ^fanconi anemia complementation group C,^  *^SUMO1P1^* ^SUMO1 pseudogene 1,^ *^ERBB3^* ^erb-B2 receptor tyrosine kinase 3^
