# Supplementary material for: Conceptualization of functional single nucleotide polymorphisms of polycystic ovarian syndrome genes: an in silico approach
Source: J Endocrinol Invest. 2021 Jan 27;44(8):1783–93. doi: 10.1007/s40618-021-01498-4 (PMC8285346; doi:10.1007/s40618-021-01498-4)
Supplement: Supplementary file 2 — Supplementary file2 Online Resource 2. Details of selected genome wide significant genes for downstream analysis (DOCX 51 KB) [file 40618_2021_1498_MOESM2_ESM.docx]

**Online Resource 2.** Details of selected genome wide significant genes for downstream analysis.

| \| **Sl. no.** \| **Gene** \| **Gene symbol** \| **SNPs** \| **Chromosome coordinates** \| **References** \| \| --- \| --- \| --- \| --- \| --- \| --- \| \| 1 \| *ADP Ribosylation Factor Like GTPase 14 Effector Protein/Follicle Stimulating Hormone Subunit Beta* \| *ARL14EP/FSHB* \| rs11031005 \| chr11:30,323,104-30,338,458 /  30,231,014-30,235,261 \| [34] \| \| 2 \| *Aminopeptidase O* \| *C9orf3 (AOPEP)* \| rs3802457 \| chr9:94,726,669-95,148,264 \| [31] \| \|  \|  \|  \| rs4385527 \|  \| [31] \| \|  \|  \|  \| rs10993397 \|  \| [32] \| \| 3 \| *DENN domain-containing protein 1A* \| *DENND1A* \| rs10818854 \| chr9:123,379,654-123,930,158 \| [30] \| \|  \|  \|  \| rs10986105 \|  \| [30] \| \|  \|  \|  \| rs9696009 \|  \| [34] \| \|  \|  \|  \| rs2479106 \|  \| [30] \| \| 4 \| *Erb-B2 Receptor Tyrosine Kinase 3/Ras-Related Protein* \| *ERBB3/RAB5B* \| rs2271194 \| chr12:56,076,799-56,103,505 /  55,973,913-55,996,683 \| [34] \| \| 5 \| *Erb-B2 Receptor Tyrosine Kinase 4* \| *ERBB4* \| rs2178575 \| chr2:211,375,717-212,538,841 \| [34] \| \|  \|  \|  \| rs1351592 \|  \| [33] \| \| 6 \| *FA Complementation Group C* \| *FANCC* \| rs7864171 \| chr9:95,099,054-95,426,796 \| [34] \| \| 7 \| *Follicle Stimulating Hormone Subunit Beta* \| *FSHB* \| rs11031006 \| chr11:30,231,014-30,235,261 \| [32] \| \| 8 \| *Follicle Stimulating Hormone Receptor* \| *FSHR* \| rs2349415 \| chr2:48,953,161-49,154,527 \| [31] \| \|  \|  \|  \| rs2268361 \|  \| [31] \| \| 9 \| *GATA Binding Protein 4/ Nei Like DNA Glycosylase 2* \| *GATA4 / NEIL2* \| rs804279 \| chr8:11,676,931-11,760,002 /  11,769,639-11,787,345 \| [32] \| \| 10 \| *High-mobility group AT-hook 2* \| *HMGA2* \| rs2272046 \| chr12:65,824,131-65,966,295 \| [31] \| \| 11 \| *Insulin Receptor* \| *INSR* \| rs2059807 \| chr19:7,112,255-7,294,414 \| [31] \| \| 12 \| *Potassium Voltage-Gated Channel Subfamily A Member 4/ Follicle Stimulating Hormone Subunit Beta* \| *KCNA4 / FSHB* \| rs11031006 \| chr11:30,009,730-30,017,030 / 30,231,014-30,235,261 \| [32] \| \| 13 \| *KRR1 Small Subunit Processome Component Homolog* \| *KRR1* \| rs1275468 \| chr12:75,490,863-75,511,636 \| [33] \| \|  \|  \|  \| rs1795379 \|  \| [34] \| \| 14 \| *Luteinizing Hormone/Choriogonadotropin Receptor* \| *LHCGR* \| rs13405728 \| chr2:48,686,774-48,755,730 \| [30] \| \| 15 \| *Microtubule Associated Protein RP/EB Family Member 1* \| *MAPRE1* \| rs853854 \| chr20:32,819,780-32,850,405 \| [34] \| \| 16 \| *Plasminogen Receptor With A C-Terminal Lysine* \| *PLGRKT* \| rs10739076 \| chr9:5,357,966-5,438,539 \| [34] \| \| 17 \| *Ras-Related Protein, Sulfite Oxidase* \| *RAB5B , SUOX* \| rs705702 \| chr12:55,973,913-55,996,683 /  55,992,547-56,006,641 \| [31] \| \| 18 \| *RAD50 Double Strand Break Repair Protein* \| *RAD50* \| rs13164856 \| chr5:132,556,019-132,646,349 \| [33] \| \| 19 \| *SUMO1 Pseudogene 1* \| *SUMO1P1* \| rs6022786 \| chr20:53,874,498-53,875,709 \| [31] \| \| 20 \| *Thyroid Adenoma-Associated Protein* \| *THADA* \| rs13429458 \| chr2:43,230,836-43,596,046 \| [30] \| \|  \|  \|  \| rs7563201 \|  \| [33] \| \|  \|  \|  \| rs12468394 \|  \| [30] \| \|  \|  \|  \| rs12478601 \|  \| [30] \| \| 21 \| *TOX High Mobility Group Box Family Member 3* \| *TOX3* \| rs8043701 \| chr16:52,436,415-52,547,802 \| [34] \| \|  \|  \|  \| rs4784165 \|  \| [31] \| \| 22 \| *Yes Associated Protein 1* \| *YAP1* \| rs11225154 \| chr11:102,109,957-102,233,424 \| [33] \| \|  \|  \|  \| rs1894116 \|  \| [31] \| \| 23 \| *Zinc Finger And BTB Domain Containing 16* \| *ZBTB16* \| rs1784692 \| chr11:114,059,576-114,256,770 \| [34] \| |  |  |  |  |  |
| --- | --- | --- | --- | --- | --- | --- | --- | --- | --- | --- | --- | --- | --- | --- | --- | --- | --- | --- | --- | --- | --- | --- | --- | --- | --- | --- | --- | --- | --- | --- | --- | --- | --- | --- | --- | --- | --- | --- | --- | --- | --- | --- | --- | --- | --- | --- | --- | --- | --- | --- | --- | --- | --- | --- | --- | --- | --- | --- | --- | --- | --- | --- | --- | --- | --- | --- | --- | --- | --- | --- | --- | --- | --- | --- | --- | --- | --- | --- | --- | --- | --- | --- | --- | --- | --- | --- | --- | --- | --- | --- | --- | --- | --- | --- | --- | --- | --- | --- | --- | --- | --- | --- | --- | --- | --- | --- | --- | --- | --- | --- | --- | --- | --- | --- | --- | --- | --- | --- | --- | --- | --- | --- | --- | --- | --- | --- | --- | --- | --- | --- | --- | --- | --- | --- | --- | --- | --- | --- | --- | --- | --- | --- | --- | --- | --- | --- | --- | --- | --- | --- | --- | --- | --- | --- | --- | --- | --- | --- | --- | --- | --- | --- | --- | --- | --- | --- | --- | --- | --- | --- | --- | --- | --- | --- | --- | --- | --- | --- | --- | --- | --- | --- | --- | --- | --- | --- | --- | --- | --- | --- | --- | --- | --- | --- | --- | --- | --- | --- | --- | --- | --- | --- | --- | --- | --- | --- | --- | --- | --- | --- | --- | --- | --- | --- | --- | --- | --- | --- | --- | --- | --- | --- | --- | --- | --- | --- | --- |

*^SNPs^* ^single nucleotide polymorphisms, chr chromosome^
