# Supplementary material for: Conceptualization of functional single nucleotide polymorphisms of polycystic ovarian syndrome genes: an in silico approach
Source: J Endocrinol Invest. 2021 Jan 27;44(8):1783–93. doi: 10.1007/s40618-021-01498-4 (PMC8285346; doi:10.1007/s40618-021-01498-4)
Supplement: Supplementary file 3 — Supplementary file3 Online Resource 3. Deleterious nsSNPs and associated amino acid change (DOCX 17 KB) [file 40618_2021_1498_MOESM3_ESM.docx]

**Online Resource 3.** Deleterious nsSNPs and associated amino acid change

| **Sl no.** | **Gene** | **Gene symbol** | **MAF** | **rsID** | **Chromosome** | **Observed alleles** | **Minor allele** | **Amino acid change** |
| --- | --- | --- | --- | --- | --- | --- | --- | --- |
| 1 | *Erb-B2 Receptor Tyrosine Kinase 4* | *ERBB4* | 0.0002 | rs192066345 | chr2 | A/G | G | Leu to Pro |
| 2 | *Erb-B2 Receptor Tyrosine Kinase 4* | *ERBB4* | 0.0002 | rs528780505 | chr2 | A/T | T | Ile to Asn |
| 3 | *GATA Binding Protein 4* | *GATA4* | - | rs180765750 | chr8 | A/G | A | Arg to His |
| 4 | *Insulin Receptor* | *INSR* | 0.0002 | rs79312957 | chr19 | A/G | A | Arg to Cys |
| 5 | *Luteinizing Hormone/Choriogonadotropin Receptor* | *LHCGR* | 0.0002 | rs121912525 | chr2 | A/C | T | Ser to Tyr |
| 6 | *Sulfite oxidase* | *SUOX* | 0.0002 | rs575660698 | chr12 | A/G | A | Arg to His |
| 7 | *Yes Associated Protein 1* | *YAP1* | - | rs199505545 | chr11 | C/G | C | Trp to Ser |

*^MAF^* ^minor allele frequency, chr chromosome^
