# Supplementary material for: Conceptualization of functional single nucleotide polymorphisms of polycystic ovarian syndrome genes: an in silico approach
Source: J Endocrinol Invest. 2021 Jan 27;44(8):1783–93. doi: 10.1007/s40618-021-01498-4 (PMC8285346; doi:10.1007/s40618-021-01498-4)
Supplement: Supplementary file 4 — Supplementary file4 Online Resource 4. Polarity and hydrophobicity/hydrophilicity of the reported deleterious nsSNPs (DOCX 15 KB) [file 40618_2021_1498_MOESM4_ESM.docx]

| **Sl no.** | **Gene** | **SNP ID** | **MAF** | **Mutation** | **Polarity** | **Change in polarity** | **Hydropathy index** | **Change in hydrophobicity/ hydrophilicity** |
| --- | --- | --- | --- | --- | --- | --- | --- | --- |
| 1 | *ERBB4* | rs192066345 | 0.0002 | L131P | Nonpolar to Nonpolar | No | 3.8 to -1.6 | Yes |
| 2 | *ERBB4* | rs528780505 | 0.0002 | I362N | Nonpolar to Polar(uncharged) | Yes | 4.5 to -3.5 | Yes |
| 3 | *GATA4* | rs180765750 | - | R283H | Polar to Polar | No | -4.5 to -3.2 | No |
| 4 | *INSR* | rs79312957 | 0.0002 | R413C | Polar to Polar (uncharged) | No | -4.5 to 2.5 | Yes |
| 5 | *LHCGR* | rs121912525 | 0.0002 | S531Y | Polar (uncharged) to Polar (uncharged) | No | -0.8 to -1.3 | No |
| 6 | *SUOX* | rs575660698 | 0.0002 | R269H | Polar to Polar | No | -4.5 to -3.2 | No |
| 7 | *YAP1* | rs199505545 | - | W177S | Nonpolar to Polar (uncharged) | Yes | -0.9 to -0.8 | No |

**Online Resource 4.** Polarity and hydrophobicity/hydrophilicity of the reported deleterious nsSNPs

*^SNP^* ^single nucleotide polymorphism,^ *^MAF^* ^minor allele frequency,^ *^ERBB4^* ^erb-b2 receptor tyrosine kinase 4,^ *^GATA4^* ^GATA binding protein 4,^ *^INSR^* ^insulin receptor,^ *^LHCGR^* ^luteinizing hormone/choriogonadotropin receptor,^ *^SUOX^* ^sulfite oxidase,^ *^YAP1^* ^yes^ ^associated protein 1^
