# Supplementary material for: Conceptualization of functional single nucleotide polymorphisms of polycystic ovarian syndrome genes: an in silico approach
Source: J Endocrinol Invest. 2021 Jan 27;44(8):1783–93. doi: 10.1007/s40618-021-01498-4 (PMC8285346; doi:10.1007/s40618-021-01498-4)
Supplement: Supplementary file 5 — Supplementary file5 Online Resource 5. Total energy (wild and mutant type), change in energy and RMSD value of the reported deleterious nsSNPs (DOCX 15 KB) [file 40618_2021_1498_MOESM5_ESM.docx]

| **Sl no.** | **Gene** | **SNP ID** | **MAF** | **Total Energy (Wild Type protein)** | **Total Energy (Mutant protein)** | **Change in Energy** | **Root Mean Square Deviation (Ǻ)** |
| --- | --- | --- | --- | --- | --- | --- | --- |
| 1 | *ERBB4* | rs192066345 | 0.0002 | -33905.453 | -33700.68 | -204.773 | 0.020 |
| 2 | *ERBB4* | rs528780505 | 0.0002 | -33905.453 | -34064.195 | 158.742 | 0.001 |
| 3 | *GATA4* | rs180765750 | - | -8568.618 | -8183.757 | -384.861 | 0.031 |
| 4 | *INSR* | rs79312957 | 0.0002 | -36870.891 | -36611.379 | -259.512 | 0.001 |
| 5 | *LHCGR* | rs121912525 | 0.0002 | -15750.735 | -15492.775 | -257.96 | 0.002 |
| 6 | *SUOX* | rs575660698 | 0.0002 | -21583.27 | -21255.203 | -328.067 | 0.005 |
| 7 | *YAP1* | rs199505545 | - | -16826.201 | -17001.199 | 174.998 | 0.003 |

**Online Resource 5.** Total energy (wild and mutant type), change in energy and RMSD value of the reported deleterious nsSNPs

*^SNP^* ^single nucleotide polymorphism,^ *^MAF^* ^minor allele frequency,^ *^ERBB4^* ^erb-b2 receptor tyrosine kinase 4,^ *^GATA4^* ^GATA binding protein 4,^ *^INSR^* ^insulin receptor,^ *^LHCGR^* ^luteinizing hormone/choriogonadotropin receptor,^ *^SUOX^* ^sulfite oxidase,^ *^YAP1^* ^yes^ ^associated protein 1^
