# Supplementary material for: Conceptualization of functional single nucleotide polymorphisms of polycystic ovarian syndrome genes: an in silico approach
Source: J Endocrinol Invest. 2021 Jan 27;44(8):1783–93. doi: 10.1007/s40618-021-01498-4 (PMC8285346; doi:10.1007/s40618-021-01498-4)
Supplement: Supplementary file 6 — Supplementary file6 Online Resource 6. miRNA target site SNPs with MAF>0.1 (DOCX 16 KB) [file 40618_2021_1498_MOESM6_ESM.docx]

**Online Resource 6.**  miRNA target site SNPs with MAF > 0.1

| Sl no. | Gene | Common SNPs among MirSNP, miRNASNP3 and PolymiRTS databases | MAF | miRNA binding at mutant allele | miRNA binding at ancestral allele | Effect |
| --- | --- | --- | --- | --- | --- | --- |
| 1 | *HMGA2* | rs1042725 | 0.41 | hsa-miR-10a-3p | - | Break |
|  |  |  |  | - | hsa-miR-4760-3p | Create |
|  |  |  |  | - | hsa-miR-664-3p | Create |
|  |  | rs7312910 | 0.29 | hsa-miR-185-5p | - | Break |
|  |  |  |  | - | hsa-miR-3125 | Create |
|  |  |  |  | - | hsa-miR-3916 | Create |
|  |  |  |  | hsa-miR-4306 | - | Break |
|  |  |  |  | hsa-miR-4419a | - | Decrease |
|  |  |  |  | hsa-miR-4510 | - | Enhance |
|  |  |  |  | hsa-miR-4644 | - | Break |
| 2 | *MAPRE1* | rs242538 | 0.19 | - | hsa-miR-28-3p | Create |
|  |  |  |  | - | hsa-miR-708-3p | Create |
|  |  |  |  | hsa-miR-4694-5p | - | Break |

*^MAF^* ^minor allele frequency,^ *^HMGA2^* ^high mobility group AT-hook 2,^ *^MAPRE1^* ^microtubule associated protein rp/eb family member 1^
