# Supplementary material for: Conceptualization of functional single nucleotide polymorphisms of polycystic ovarian syndrome genes: an in silico approach
Source: J Endocrinol Invest. 2021 Jan 27;44(8):1783–93. doi: 10.1007/s40618-021-01498-4 (PMC8285346; doi:10.1007/s40618-021-01498-4)
Supplement: Supplementary file 7 — Supplementary file7 Online Resource 7. Impact of SNPs in the transcription factor binding site with MAF>0.1 (DOCX 17 KB) [file 40618_2021_1498_MOESM7_ESM.docx]

**Online Resource 7.** Impact of SNPs in the transcription factor binding site with MAF > 0.1

| Sl no. | Gene name | rsID | Allele | Function | MAF | Generated sites | Deleted sites |
| --- | --- | --- | --- | --- | --- | --- | --- |
| 1 | *NEIL2* | rs8191511 | C>T | upstream | 0.12 | FHXB, PROP1, EN1, LMX1B, ATATA, E4BP4, LHX5, BRN4, XFD1, TEF, PROP1 | SOX21, PDX1, CHR, OCT2 |
| 2 | *NEIL2* | rs8191514 | A>C/G | upstream | 0.13 | MAZ, ZBTB7, SP4, EGR2, ZNF281, CTCF, EGR1, ZBP89, PURALPHA, ZNF219, INSM1, ZBED4, ZNF37A, EKLF, SPZ1, NXF/ARNT, CREB3L2, ATF6, HAS, MIZ1 | EKLF, SPZ1 |
| 3 | *GATA4* | rs61277615 | C>T | 5′-UTR | 0.20 |  | PLAG1, ZNF704, HDBP1, ZNF704 |
| 4 | *DENND1A* | rs62579216 | C>T | upstream | 0.20 | PAX2, PREB | PATZ1, IKLF, ZTRE, E2F1, ZBTB7, ZBED4, SP1, EGR1, ZFX |
| 5 | *SUMO1P1* | rs6068700 | C>G/T | upstream | 0.21 | VMYB, HMX2, MYT1, ZNF384 | PPARG, GKLF, ZNF384 |
| 6 | *INSR* | rs2860189 | A>C/T | upstream | 0.26 | NXF/ARNT, PLAG1, STAT1 | ZIC3 |
| 7 | *INSR* | rs1864009 | A>C | upstream | 0.39 | WT1, EGR1, NRF1, AHRARNT, ZF5, HELT, ZSCAN10, ZF5 | KAISO |
| 8 | *ARL14EP* | rs1222220 | A>C/T | upstream | 0.26 | OCT1, P53 | ATBF1, P53 |
| 9 | *ZBTB16* | rs11214857 | A>G | upstream | 0.34 | CTCF, PLAG1, ZBP89, PLAG1, ZBED4, KKLF, RREB1, TIEG, EGR2, KLF2, IKLF, MAZ, NM23, SALL2, ZFP410, SP1, SREBP, MTF-1 | ZNF444 |
| 10 | *ERBB3* | rs7297175 | C>T | upstream | 0.34 | MZF1, RREB1, PEGASUS | FAST1 |

*^MAF^* ^minor allele frequency,^ *^NEIL2^* ^nei like dna glycosylase 2^*^, GATA4 GATA Binding Protein 4, DENND1A^* ^DENN Domain Containing 1A,^ *^SUMO1P1^* ^SUMO1 pseudogene 1,^ *^INSR^* ^insulin receptor^*^, ARL14EP^* ^ADP ribosylation factor like GTPase 14 effector protein,^ *^ZBTB16^* ^zinc finger and BTB domain containing 16,^ *^ERBB3^* ^erb-B2 receptor tyrosine kinase 3^
