# Supplementary material for: Conceptualization of functional single nucleotide polymorphisms of polycystic ovarian syndrome genes: an in silico approach
Source: J Endocrinol Invest. 2021 Jan 27;44(8):1783–93. doi: 10.1007/s40618-021-01498-4 (PMC8285346; doi:10.1007/s40618-021-01498-4)
Supplement: Supplementary file 8 — Supplementary file8 Online Resource 8. SNPs in enhancers and their altered regulatory motifs with MAF>0.1 (DOCX 16 KB) [file 40618_2021_1498_MOESM8_ESM.docx]

**Online Resource 8.** SNPs in enhancers and their altered regulatory motifs with MAF > 0.1

| Sl no. | Gene | Chromosome | rs ID | MAF | Reference Allele | Alternative Allele | Enhancer ID | Functional annotation | Regulatory motifs altered |
| --- | --- | --- | --- | --- | --- | --- | --- | --- | --- |
| 1 | *DENND1A* | chr9 | rs12237685 | 0.36 | T | A,C | enh25987 | intronic | En-1 |
| 2 | *C9orf3* | chr9 | rs6479596 | 0.13 | A | G | enh25789 | intronic | PLZF |
| 3 | *INSR* | chr19 | rs73488786 | 0.1 | C | A | enh17891 | intronic | AP-1, BDP1, CTCF, SMC3 |
| 4 | *INSR* | chr19 | rs11670022 | 0.14 | C | T | enh17891 | intronic | E2A, HEN1, Lmo2, Myf, ZEB1 |
| 5 | *KRR1* | chr12 | rs12815116 | 0.23 | C | A | vista14045 | intronic | Maf, Nkx3, Sox |
| 6 | *RAD50* | chr5 | rs56394135 | 0.12 | C | T | enh22568 | intronic | Dbx2, Maf, Pou2f2, THAP1 |
| 7 | *RAD50* | chr5 | rs13176348 | 0.14 | C | T | enh22569 | intronic | E2F, Pou2f2 |
| 8 | *RAD50* | chr5 | rs3846732 | 0.4 | T | C | enh22570 | intronic | GR, SPIB |

*^MAF^* ^minor allele frequency,^ *^chr^* ^chromosome,^ *^DENND1A^* ^DENN domain containing 1A,^ *^C9orf3^* ^Chromosome 9 open reading frame 3,^ *^INSR^* ^Insulin Receptor,^ *^KRR1^* ^KRR1 small subunit processome component homolog,^ *^RAD50^* ^RAD50 double strand break repair protein^
